# The Paris Dental Congress

**Published:** 1900-10-15

**Authors:** 


### THE PARIS DENTAL CONGRESS.

Reprinted from *The Dental Record*, of London.

The Third International Dental Congress, convened in Paris on August 8th and continued in session until the 14th ulto., will go down into history as one of the interesting epochs of the profession. The selection of Paris as a place of meeting during this busy year may not have been the wisest one, for while the Exposition may have proved an incentive to a few who otherwise would not have been in attendance at the congress, there is but little doubt that many were deterred from being present on this account. Be this as it may, the congress was a success from beginning to end, and a happy combination of favorable circumstances brought about a result of which the profession should be justly proud. When it is taken into consideration that nearly all in dentistry in the way of scientific advancement and practical work has been confined to the century now so rapidly drawing to a close, and a goodly portion of it to the latter half of this limited period, the results of such a meeting as that which has just taken place must be, to say the least, extremely gratifying. It is to be deplored that a better place of meeting was not secured, and that the clinics, papers, discussions and exhibits were not condensed under one roof. As it was, much valuable time was spent in going from place to place, and in this way many of the delegates were denied the privilege of seeing and hearing those things in which they were most interested. But it is not our intention to criticise the management of the congress; only praise is due to the gentlemen who so faithfully labored to bring about such a successful meeting.

The opening session of the congress was held within the Exhibition grounds, in the Grand Salle of the Palais des Congres, with Professor M. C. Brouardel, member of the Faculty of Medicine of Paris, as honorary president, and M. Gariel, professor of the Faculty of Medicine of Paris, in the capacity of acting president. Seated about these central figures were members of the various national and local committees, together with many of the more noted representatives from foreign nationalities. Considering the early hour at which the formal opening would take place, the spacious hall was well filled with an audience remarkable for its good looks, its undivided interest, and the variety of nationalities represented. Here were assembled men from all parts of the world, who had come to represent their country as delegates to this meeting. The session was formally opened with an address by M. Godon, whose remarks, continued at some length, were frequently interrupted by applause. He spoke of the official recognition of the congress by an officer of the French Government, and extended a hearty welcome to the representatives of the various foreign governments, schools and societies present. The speaker claimed that the dentist stood on an equality with the physician and surgeon, for it is he who has charge of the common entrance of the human organism, and not infrequently he it is who first discovers some approaching disturbance of the economy, and by his skill and care prevents or checks the advance of what might otherwise have been a serious malady. By his modern method he restores to usefulness those parts under his care which for a time have lost their functional worth, and in this way again steps in as a helper to the physician when other structures and organs are pathologically involved. He dwelt upon the interest of continuen scientific research, and was lavish in his praise of those who in the past had devoted so much of their time and energy in this way.

In closing he referred to the willingness of the dentist of the present day to render services to the poor, and he hoped for the establishment by the Government of a means by which this can be more fully carried out. Other officers of the congress followed M. Godon, the international secretary, M. Viau, furnishing a good idea of the healthful condition of the organization by reporting that over 1,150 names were at the present time enrolled; this appears as an increase of nearly 300 over the membership at the time of the last international meeting. After the various official communications had been received, the audience was well entertained by short addresses by representative men from the various nationalities. Mr. Aguilar spoke at some length for Spain; M. Baruch for Belgium; Dr. A. W. Harlan, of Chicago, the national chairman for the United States, responded for his country; Mr. Brunton, of Leeds, for England; Dr. Hesse for Germany; and Dr. Frank for Austria. Each one of these gentlemen delivered his remarks in his native language, the attention was most profound, and at the close of the meeting all were impressed with the fact that they had before them in the congress, thus formally opened, a rare intellectual feast.

The program for the afternoon called for an assembling of delegates at 1:30, when papers and discussions were the order of the hour. The paper by Mr. C. S. Tomes, on "The History of the Dentinal Fibril," was passed; and Dr. Hesse, of Leipsic, read a short communication describing a method of finding the movement of the lower jaw by means of a fixed plumbago point attached to the lower teeth. Mr. Mummery read a paper on "Leptothrix Racemosa," an organism occurring in great abundance in the mouth, but which had been generally overlooked by investigators. He thought it played a more important part than the cocci and bacilli, to which much greater attention had been paid. The greater portion of the afternoon was taken up by a thorough discussion on the general subject of orthodontia. The speaker, Dr. Case, is deserving of much credit for his admirable methods and unequaled results in this class of work. The paper was illustrated by lantern projections, showing in nearly every instance a picture of the plaster mask of the case before and after operation, and not a few of the appliances for bringing about the results were shown. The paper was principally confined to the facial deformities so frequently met with, and having as their cause some protrusion or retraction of the incisor and bicuspid teeth. He claimed that in very many instances a chin pronounced as being unduly prominent, was, in fact, perfectly normal, the defect being produced by a lack of prominence in the upper teeth. The speaker also claimed that while only a small portion of the face is capable of being influenced by a dental deflection, that nevertheless the mouth and lip are so directly interested in creating the facial contour and expression, that frequently a very slight deviation in these parts wrought havoc to the entire physiognomy.

The clinics were held in the rooms of two of the local dental schools, neither one of them offering sufficient space to accommodate the very great number of clinicians. The morning hours were devoted to these practical demonstrations, and every one, although recognizing the fact that there appeared to be really little that was new, showed a willingness and appeared to be specially anxious to profit by that which was being shown. The first clinics were given on Thursday morning, and while they consisted of almost every special branch of operative or mechanical procedure, special attention was devoted to those demonstrations properly considered under the former classification. Dr. Schrein exhibited some novel and thorough aseptic methods in root treatment, dwelling upon the necessity of great care and decision in the management of all root canals, either in those cases in which the pulp had but recently lost its vitality, or where after the lapse of time the contents of the canal had become more or less putrescent. This operator advocated especially the value of perfect dehydration of the pulp chamber and canals so far as possible at the first sitting, and continuing them in this state from that time until the final operation.

Dr. Herbst exhibited a number of instruments specially adapted to his methods of manipulating gold, and interested a good sized audience for nearly two hours with his operative technique, which consisted not only of his manner of working gold, but also many valuable adjuncts, each of which by itself might appear of little importance, but when taken collectively would prove of inestimable value. Those who missed seeing Dr. Herbst at work should take advantage of the earliest opportunity of seeing him. He always has something to impart, and he gives it forth in such a manner that his ideas are readily understood.

Mr. W. Hern, of London, exhibited his method of securing a perfect adaptation between the ferrule or metallic basal portion of a so-called Richmond crown and the free end of the root to which it is desired to make the attachment. In this work the clinician preferred to use the partial band, i.e., involving only the palatal or lingual and approximal sides of the root, leaving the labial side free, and so grinding and adjusting the porcelain face that it will serve as a continuation of the metal band and at the same time pass beneath the free margin of the gum, and thus avoid the unsightly appearance easily occasioned by continuing the bands entirely around the root. To obtain a perfect adaptation a temporary metallic form was employed the pin of which entered the root and the circular base plate attached to the same approximately fitted the end of the root, upon this a body of gutta-percha was placed and, after being softened by heat, was forced into position, and a perfect imprint of the root and gum was thus obtained. A plaster model from this was used upon which to construct the remainder of the appliance.

Dr. Schwartz demonstrated the use of his method of ascertaining the exact movements of the lower jaw, which was almost similar or perhaps the same as that described by Dr. Hesse already spoken of.

Dr. Tomaskee, who did not appear as a clinician, gave a description and a demonstration of his system of obtaining an impression of the mouth with a very high vault, and his method appealed to those who were fortunate enough to hear him as being of very great service. A piece of heavy copper wire, almost a quarter of an inch thick and about six inches in length was bent at right angles to serve as a handle for an impression tray or plate, which was afterward attached to it. This tray, made of a flat piece of copper or German silver plate, was cut to the shape of the roof of the mouth; that is, to approximately fit against the sides of the vault some distance below its deepest portion. When completed the plate was left flat and had a shape somewhat like the letter U with a solid center. Upon this plate a body of plaster was placed and carried into position in the high, inaccessible part of the palate. After the plaster had hardened the handle of the improvised appliance, which was removable, was withdrawn, leaving the flat metal plate and plaster in the mouth. This was left in position while the major portion of the impression was taken, which was done in the usual way, and the complete impression, now in two pieces, was removed and fitted together out of the mouth.

On Thursday afternoon the audience assembled in the general meeting had the pleasure of hearing Dr. Leon Williams and of seeing some lantern slides descriptive of his theories in regard to the fibers found in the enamel. The speaker showed the presence of fibers of some character in sections of enamel taken from some of the lower animals, but did not appear to be able to account for their presence, nor was he positive in regard to their character.

Dr. A. Howard Thompson, of Kansas, contributed a very interesting paper on the fifth tubercle of the second lower molar. The author claims that the teeth, to a greater extent than any other part of the human organism, furnishes the information as to the character and habits of the animal to which they belong, and by this means many biological problems have been solved. While this is true of all the molars, it is especially true of those confined to the lower jaw, and especially to the second molar. The fifth cusp of this tooth appears and disappears, always being present in the monkey and the lower classes of human kind. A striking characteristic, and one of considerable interest, is the fact that this cusp has always been wanting, or but slightly developed, in civilized people of all ages.

Mr. J. Lefevre reported a case of fractured lower maxilla in a man of seventy, the chief interest being the age of the patient, and the result accomplished. The bone was completely fractured at one point, and partially fractured in two others. After obtaining an approximate impression and cast, a retaining appliance was constructed consisting of heavy gold wires and bands carefully adapted and adjusted to the plaster reproduction. This when transferred to the mouth so forced the parts into position that they were almost immediately restored to usefulness.

At the Friday morning clinic Mr. Brunton demonstrated a special method of flasking for vulcanite work by the use of a specially constructed flask. The case may be said to have been flasked "on end," i.e., the flask was so constructed that the front of the flask, or that in which the anterior teeth usually rest, served as its base. When the case was inserted in the first section of the flask, the teeth were so enveloped that they did not leave their investment when the flask was opened. That portion of the flask which was to represent the gum was supplied with this material when waxing up, leaving only the palatal portion or main part of the plate exposed. The directions of the force supplied in the closing of the flask was such that little or no pressure was brought upon the frail wall of the alveolus, and the danger of cracking the palatal portion of the cast was reduced to a minimum.

Dr. Brirgotti exhibited two or three cases of implantation and transplantation, some of which having been but recently performed, and others being of several years' standing. One of the cases operated upon but a few days previously was shown, and it was found that the tooth, an upper cuspid, which had been inserted to take the place of a broken-down, diseased root, had already become quite firm, and the patient had experienced no inconvenience since having the operation performed.

A number of clinics were given on porcelain work, among the operators being Drs. Jenkins, Head and Good, the two former directing their attention to inlays and the latter to porcelain bridge work. In connection with these clinics more or less discussion was brought out in regard to the preference for a high or low fusing body, and the proper metal to use for the matrix, and, while the high fusing body was strongly favored, there appeared to be a diversity of opinion as to the use of gold or platina for the matrix, with the former as a slight favorite.

In the third general assembly M. L. Richard-Chauvin presented a communication which considered a new method of obturation by means of hard porcelain blocks. In this paper it was claimed that when used for such purposes porcelain possessed the advantages of conductibility, a fact always to be desired where mucous membranes are to be covered, also ready adaptibility, simplicity of manipulation, serviceability, cleanliness and comparative inexpensiveness. It was also argued that the work could be performed in the operating room, the adaptation of the parts and fusing of the porcelain being accomplished with the patient in the chair.

In section Dr. Griswold presented a practical paper on the "Construction of Artificial Teeth and Moveable Sets." For many years the author had practised this plan of making removable bridge-plates, his general method being that of first making a movable crown which is attached to a fixed point on two or more roots. The supporting substance, the result of numerous experiments, is an alloy possessed of sufficient elasticity to accommodate the occlusion and of enough strength to provide durability. The advantages of thus constructing a partial denture were, first, that it affords by its easy removal a means of thorough cleansing, ready repair, and additions to provide for further shrinking of the soft tissues. In addition to these advantages the principles thus designated when applied to the upper jaw possess the advantage apparent in all fixed bridges, that of an unencumbered palatal vault. Furthermore, it was argued that appliances partly supported in this way would afford a sense of security to the wearer not to be obtained when a suction plate was used.

The "Eruption of the Teeth" was the title of a paper by Mr. T. E. Constant, and it dealt principally with the many existing theories in regard to the cause of this phenomenon. Among these theories were mentioned those which considered the elongation of the roots of the teeth responsible for their eruption, a mechanical force such as might be produced by the continual opening and closing of the jaws, thus in a manner jarring the teeth to the surface by the contraction of the walls of the tooth sac, which being attached to the surface would force the teeth in that direction, and by a body of bone forming about and beneath their developing roots pushing them forward. In contradiction, very many reasons were given why none of these reasons could be accepted, and the author offered the following as an additional speculation covering the subject, viz., between the tooth and its growing walls there appears a vascular network, the pressure exerted by which is actively responsible in a mechanical way for forcing the tooth through the gum.

Monday's clinics were numerous, interesting, and instructive. Dr. Herbst showed his method of using gold and amalgam in combination, also gold and porcelain in combination. Dr. R. Weiser demonstrated a valuable method for the treatment of old or new alveolar abscess, which consisted in opening up the part by a crucial incision of the gum tissue immediately over the apex of the root of the affected tooth, and if a fistulous track was present this was slightly enlarged and the end of the root more or less exposed. This once accomplished, the absess was mechanically removed and appropriate dressings applied directly to the previously diseased parts. When no track existed through the bone, one was made with a specially adapted spear-shaped drill and the treatment conducted in the manner already mentioned. Several clinics pertaining to implantation and transplantation were delivered, each of the operators claiming for this practice the greatest amount of success. Further demonstrations were also given in the varied uses to which ceramics may be applied in dentistry as a means of reproduction in edentulous cases for the making and adaptation of gum blocks formed about single teeth, for the ready adaptation of a porcelain tooth to a certain part when more bulk is required and so forth. All of these being made possible and practical by the introduction of the electric furnace.

One of the best communications of the last session was that offered by Dr. Hinkens, who, for some time had had under consideration the examination of dental cements, and his paper, which was not read in full, showed great care and labor in its preparation. He claims that all cements for dental purposes must be mixed in exact proportion, i.e., a given amount of liquid must always be employed to be associated with a given amount of powder, and that the value of any cement will be destroyed and soon acted upon by the oral secretions unless some method is adopted by which such precision can be reached. This could possibly be obtained if it were not for the varied temperatures which seems to make it almost an impossibility.

Reports of Roentgen Ray work in dentistry were made by Dr. Bonchacourt who spoke of the advantage of unipolar excitation of the Crooke's tube, claiming that in this way both patient and operator feel free from the mistrust which formerly accompanied the use of Roentgen Rays. The examination may be made through the cheeks, or what is still better, through the gum and walls of the alveolous direct. Dr. W. A. Price gave a practical demonstration of what may be accomplished by the employment of the Roentgen Rays, and showed the results of his labor by very many valuable lantern slides. As to the practical field for this new discovery in dentistry very little was said.

The evenings were enlivened by banquets and receptions which seem to have been much appreciated, and everyone attending the congress voted it a great success, the courtesy and hospitality of our French brethren leaving nothing to be desired.

